# Artificial Intelligence Applications in the Prediction and Management of Pediatric Asthma Exacerbation: A Systematic Review

**DOI:** 10.7759/cureus.92491

**Published:** 2025-09-16

**Authors:** Fatima Mahmoud Osman Mohmed, Wafa Elrasheed Osman Homaida, Yousra Bala Babkir Abd Alla, Razan Mohamed Elahdab Hassan, Salma Hassan Mahmoud Ali, Gehad Suliman Eltayeb Elfaki Ahmed, Eitadal Ali Al Balal Abdelbagi, Manar Haider Sidahmed Elsaid

**Affiliations:** 1 General Medicine, Sharourah General Hospital, Sharourah, SAU; 2 Paediatrics, Altnagelvin Hospital, Londonderry, GBR; 3 Children’s Emergency Department, Barking, Havering and Redbridge University Hospitals NHS Trust, London, GBR; 4 Paediatrics, Faculty of Medicine, University of Khartoum, Khartoum, SDN; 5 Obstetrics and Gynaecology, Najran Armed Forces Hospital, Ministry of Defense Health Services, Najran, SAU; 6 Paediatrics, Dariyah General Hospital, Al Qassim, SAU; 7 Paediatrics/Neonatal Intensive Care Unit, Nizwa Tertiary Hospital, Nizwa, OMN; 8 Paediatrics, King Salman Armed Forces Hospital, Tabuk, SAU

**Keywords:** artificial intelligence, asthma exacerbation, machine learning, pediatric asthma, predictive modeling, systematic review

## Abstract

Pediatric asthma exacerbations remain a significant global health challenge due to their unpredictable nature and potential for severe morbidity. While artificial intelligence (AI) shows promise in improving prediction and management, the evidence base is fragmented. This systematic review synthesizes current literature on AI applications for pediatric asthma exacerbation prediction and management, evaluating model performance, clinical utility, and methodological quality. Following Preferred Reporting Items for Systematic Reviews and Meta-Analyses (PRISMA) 2020 guidelines, we searched PubMed, Scopus, Elsevier, Web of Science, and Excerpta Medica Database (Embase) (2020-2025) for studies applying AI/machine learning (ML) to pediatric asthma exacerbations. Eight studies met the inclusion criteria after screening 431 records. Data were extracted on study design, AI models, input features, outcomes, and performance metrics. Risk of bias was assessed using Risk Of Bias In Non-randomized Studies of Interventions (ROBINS-I) for non-randomized studies and the Cochrane Risk of Bias 2 (RoB 2) tool for randomized trials. Eight studies demonstrated AI’s effectiveness in predicting pediatric asthma exacerbations, outperforming traditional methods. Performance varied, with multimodal data yielding the best results. Some models faced limitations from data biases or small samples. Most studies had a low risk of bias. AI showed potential to improve clinical workflows, but real-world impact needs more research. AI shows strong potential for pediatric asthma exacerbation prediction, particularly with multimodal data. Key challenges include algorithmic bias mitigation, prospective validation, and standardization of outcome metrics. Future research should prioritize equitable model development and clinical integration.

## Introduction and background

Asthma is the most common chronic respiratory disease in children, affecting millions globally and representing a leading cause of emergency visits, hospital admissions, and school absenteeism [1]. Pediatric asthma exacerbations, acute deteriorations in respiratory status requiring intervention, are especially concerning due to their unpredictable onset, rapid progression, and potential for severe morbidity [2]. Despite the availability of effective pharmacological therapies and adherence to established clinical guidelines, preventing these episodes remains challenging [3]. The triggers for exacerbations are multifactorial, encompassing environmental exposures, respiratory infections, allergen sensitization, and psychosocial stressors, often interacting in complex ways that make risk prediction difficult [4]. Conventional approaches such as symptom diaries, peak expiratory flow monitoring, and clinical risk scores provide useful information but are hindered by recall bias, incomplete data capture, and limited ability to identify subtle physiological changes before symptoms escalate [5].

Artificial Intelligence (AI), including machine learning (ML) and deep learning (DL) methods, offers new opportunities to address these challenges by processing large, heterogeneous datasets and identifying non-linear relationships beyond the reach of traditional statistical methods [6]. In pediatric asthma, AI systems can integrate multimodal inputs such as electronic health records (EHRs), spirometry results, wearable sensor outputs, environmental pollutant data, and patient-reported outcomes to generate individualized risk profiles [7]. Predictive algorithms, ranging from decision trees and ensemble models to recurrent neural networks, have demonstrated promising performance in forecasting exacerbations, optimizing medication use, and supporting real-time disease monitoring [8]. Beyond prediction, AI-driven decision support tools could guide timely clinical interventions, reduce hospitalizations, and improve quality of life by enabling proactive rather than reactive asthma care [9].

Although a growing body of research has explored AI’s role in predicting and managing pediatric asthma exacerbations, the evidence base remains fragmented. Existing studies vary in data sources, patient populations, algorithmic approaches, and outcome definitions, with inconsistent reporting of validation strategies, interpretability, and comparisons to standard care. This variability makes it difficult to assess the true readiness of AI tools for clinical adoption. A systematic synthesis of these studies is therefore essential to identify current capabilities, highlight methodological strengths and weaknesses, and map gaps for future investigation. This review aims to critically appraise the literature on AI applications for pediatric asthma exacerbation prediction and management, focusing on model characteristics, input features, predictive performance, interpretability, and potential for real-world integration.

## Review

Methodology

Search Strategy

This systematic review was conducted in accordance with the Preferred Reporting Items for Systematic Reviews and Meta-Analyses (PRISMA) 2020 guidelines to ensure methodological rigor and transparency [10]. A comprehensive literature search was performed across five major electronic databases: PubMed, Scopus, Elsevier, Web of Science, and Excerpta Medica Database (Embase). The search strategy combined controlled vocabulary (e.g., Medical Subject Headings (MeSH) terms) and free-text keywords related to “Artificial Intelligence”, “Machine Learning”, “Deep Learning”, “Pediatric Asthma”, and “Asthma Exacerbation”. Boolean operators (“AND,” “OR”) were applied to refine the search, and database-specific filters were adjusted accordingly to maximize retrieval. Only studies published in the last five years (2020-2025) were included, as this time frame was deemed critical to capture the most recent technological advancements and clinically relevant AI applications, given the rapid pace of progress in computational methods. Reference lists of included studies and relevant reviews were also manually screened to identify additional eligible articles.

Eligibility Criteria

Studies were selected based on predefined eligibility criteria. We included peer-reviewed original research articles that applied AI-based models, such as ML, DL, or hybrid approaches, for the prediction or management of pediatric asthma exacerbations. Eligible studies had to involve participants aged 18 years or younger and report relevant performance metrics of AI models. Only articles published in English were considered to ensure accuracy in data extraction and interpretation. Exclusion criteria comprised studies focused solely on adult populations, articles not involving AI methods, conference abstracts without full text, review papers, editorials, and commentaries. Studies not reporting sufficient methodological or performance details for AI models were also excluded.

Study Selection and Data Management

All search results from the databases were exported into EndNote X9 reference management software (Clarivate Plc, Philadelphia, Pennsylvania, United States) for organization and duplicate removal. Two independent reviewers screened titles and abstracts for relevance, followed by a full-text review of potentially eligible studies. Disagreements at any stage were resolved through discussion or, if necessary, consultation with a third reviewer. This process ensured unbiased selection and adherence to inclusion criteria.

Data Extraction

A standardized data extraction form was developed to collect key information from the included studies. Extracted data included study characteristics (country, year, design, and sample size), patient demographics, AI model type, input features used, predicted outcome, performance metrics, validation methods, comparison with traditional approaches, and interpretability/explainability aspects. Extraction was performed independently by two reviewers to minimize errors and ensure consistency.

Risk of Bias Assessment

The methodological quality and risk of bias for the included studies were evaluated using appropriate tools based on study design. For non-randomized studies, the Risk Of Bias In Non-randomized Studies of Interventions (ROBINS-I) tool was applied, assessing domains such as confounding, participant selection, and outcome measurement [11]. For randomized controlled trials, the Cochrane Risk of Bias 2 (RoB 2) tool was used, covering randomization, deviations from intended interventions, missing outcome data, and selective reporting [12]. Risk of bias assessments were independently performed by two reviewers, with consensus reached through discussion.

Data Synthesis

Given the expected heterogeneity in AI model types, input features, outcome definitions, validation strategies, and performance metrics, a meta-analysis was not conducted. Pooling quantitative results was deemed inappropriate because variations in algorithms, datasets, and study populations could lead to misleading conclusions. Instead, findings were synthesized narratively, with results organized into thematic categories reflecting model characteristics, predictive accuracy, interpretability, and clinical applicability.

Results

Study Selection

**Figure 1:**
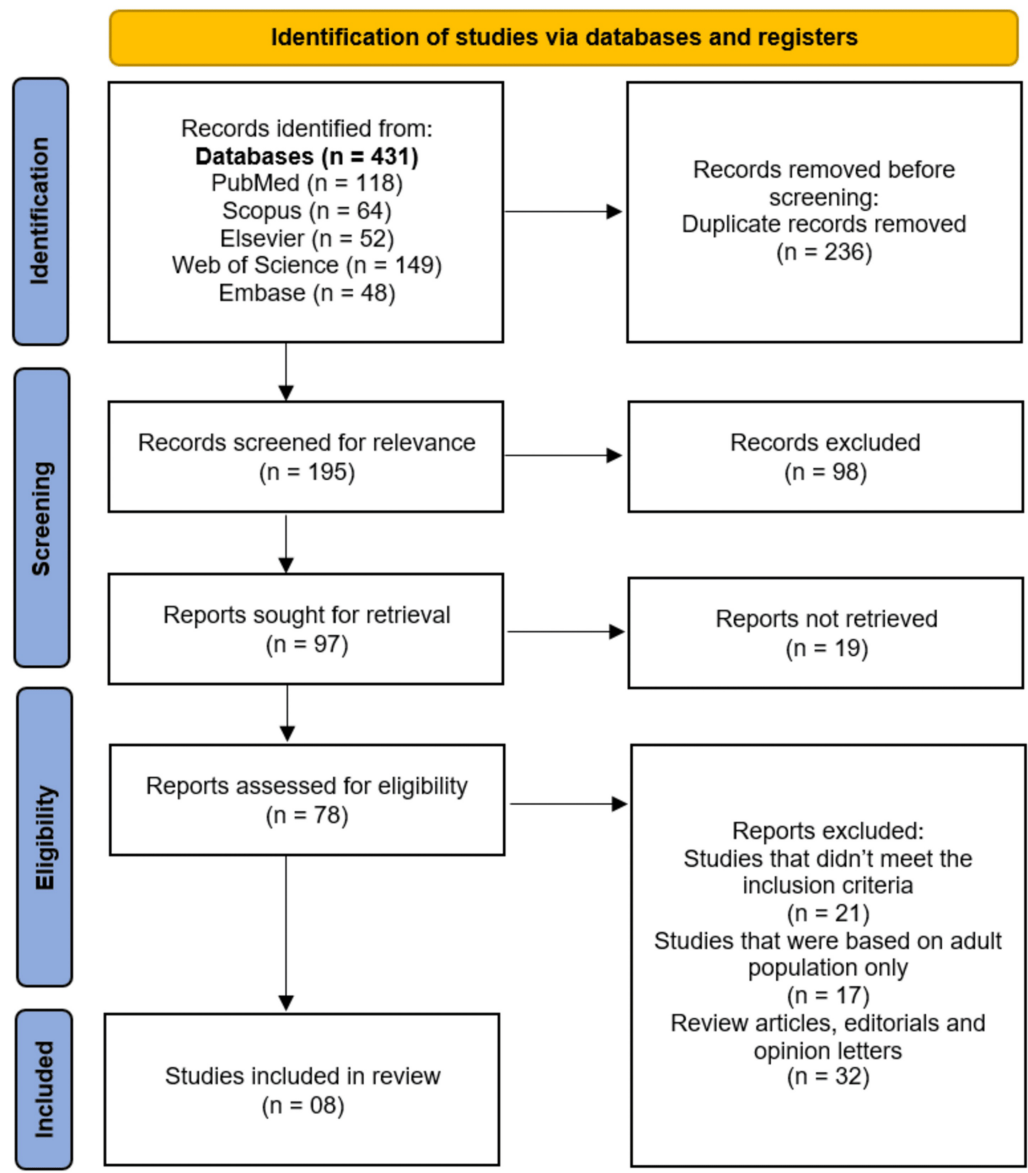
Study selection process on PRISMA flowchart PRISMA: Preferred Reporting Items for Systematic Reviews and Meta-Analyses

A total of 431 records were retrieved from the five databases: 118 from PubMed, 64 from Scopus, 52 from Elsevier, 149 from Web of Science, and 48 from Embase. After removing 236 duplicate records, 195 studies were screened for relevance. Of these, 98 records were excluded based on title and abstract screening. The remaining 97 reports were sought for full-text retrieval, of which 19 could not be accessed. A further 70 reports were excluded after full-text assessment for the following reasons: studies that did not meet inclusion criteria (n = 21), studies focusing exclusively on adult populations (n = 17), and review articles, editorials, or opinion letters (n = 32). Eight studies were included in the final systematic review [13-20]. Figure 1 shows the PRISMA flowchart.

Characteristics of Included Studies

The systematic review included eight studies that investigated the application of AI in the prediction and management of pediatric asthma exacerbations [13-20]. The studies were conducted across multiple countries, with the majority (n=6) originating from the United States [14-20], and one from Poland [13] (Table 1). The study designs varied, including retrospective cohort studies (n=4) [15,16,18,20], observational studies (n=2) [13,14], a randomized clinical trial [19], and a model development study [17]. Sample sizes ranged widely, from 149 participants in a home-monitoring study [13] to 24,283 in a retrospective analysis of prehospital pediatric asthma encounters [14]. The age groups primarily focused on pediatric populations, with some studies including adults for comparative analysis [13].

**Table 1:** Characteristics of included studies PEF: peak expiratory flow; AI: artificial intelligence; ML: machine learning; MLP: multilayer perceptron; AUC: area under the curve; SES: socioeconomic status; CDS: clinical decision support; SDOH: social determinants of health; EHR: electronic health record; AE: adverse event; A-GPS: assisted-global positioning system; RF: random forest; LR: logistic regression; HOUSES: Housing-Based Socioeconomic Status; IQR: interquartile range

Author(s)	Country	Study Design	Sample Size (n)	Population/Age Range/Mean Age	Data Source	AI Model Used	Asthma Outcome Predicted/Managed	Validation Method	Main Findings
Emeryk et al., [13] (2023)	Poland	Observational (6-month)	149 (90 children, 59 adults)	Children and adults	AI-aided home stethoscope, SpO2 meter, PEF meter, health state survey	ML models	Prediction of asthma exacerbations	AUC	Best single-parameter predictors: wheezes for young children, rhonchi for older children, survey answers for adults. Combining parameters yielded highest efficacy.
Harmon et al., [14] (2025)	United States	Retrospective Observational	24,283	2–18 years	ESO Data Collaborative (2018–2021)	MLP	Identification of pediatric asthma exacerbation	50/50 train-test split; 90/10 internal validation	MLP model showed highest performance; ML models outperformed rule-based models.
Hurst et al., [15] (2022)	United States	Retrospective cohort study	5982	Pediatric population	EHR + spatial and temporal environmental data	xgBoost	Prediction of asthma exacerbation (30–180 days)	Model performance evaluated using AUC	EHR-only models had moderate performance. Adding spatial data didn’t improve performance. Longer timeframes more useful.
Juhn et al., [16] (2022)	United States	Retrospective Cohort Study / Case Study	Not specified (small sample)	Pediatric	EHRs; SES measured by HOUSES index	Existing ML Models	Asthma Exacerbation Prediction	Comparison of balanced error rate across SES groups	Predictive performance was worse in low-SES children; lower SES linked to more missing EHR data, suggesting data incompleteness may drive AI bias
Overgaard et al., [17] (2022)	United States	Model development study	Not reported	Pediatric patients	Structured data & unstructured clinical notes from patient records	ML model	Prediction of asthma exacerbation risk	AUC = 0.8	ML-based CDS system predicted asthma exacerbation with good performance; model emphasized contextual data, usability, and explainability.
Rezaeiahari et al., [18] (2024)	United States	Retrospective cohort study	22,631	5–18 years	Arkansas All-Payer Claims Database (Medicaid data)	Conditional RF	Asthma-related hospitalizations and ED visits (2019)	Out-of-bag validation; training/test performance reported	Moderate accuracy; prior year hospital/ED visits and medication use were key predictors; SDOH variables not impactful.
Seol et al., [19] (2021)	United States	Randomized Clinical Trial (Pragmatic, Single-center)	184	Median: 8.5 years	EHRs	ML-based (A-GPS system)	Prediction of asthma exacerbation; Support in asthma management	Randomization; Control vs. Intervention group comparison	A-GPS reduced clinician time reviewing EHRs; No significant difference in AE frequency; Suggested efficiency in care.
Sills et al., [20] (2021)	United States	Retrospective Cohort	9,069	Median: 6 years (IQR: 4–10)	EHRs (5 EDs in 1 system)	AutoML (with benchmarking: RF, LR)	Need for hospitalization (ED disposition)	80/20 Train-Test Split; AUC, Accuracy, F1	AutoML achieved higher AUCs than RF and LR; improved prediction of hospital admission.

AI models employed in these studies included ML algorithms such as multilayer perceptron (MLP) [14], xgBoost [15], conditional random forest [18], AutoML [20], and ML-based clinical decision support (CDS) systems [17,19]. Data sources were diverse, encompassing electronic health records (EHRs) [15-17,19,20], prehospital emergency medical services (EMS) data [14], home monitoring devices (e.g., AI-aided stethoscopes) [13], and administrative claims databases [18]. The primary outcomes measured were asthma exacerbation prediction [13-18,20] and management support [19]. Validation methods included area under the receiver operating characteristic curve (AUC) [13,15,17,18,20], F1 scores [14], and balanced error rates (BER) [16].

Performance of AI Models

The performance metrics of the AI models demonstrated varying levels of efficacy in predicting and managing pediatric asthma exacerbations (Table 2). Emeryk et al. reported high predictive accuracy for asthma exacerbations using an AI-aided stethoscope, with AUCs of 84% for wheezes in young children, 81% for rhonchi in older children, and 92% for survey responses in adults [13]. Combining multiple parameters further improved performance, highlighting the utility of multimodal data integration.

**Table 2:** Performance metrics of AI models PEF: peak expiratory flow; AI: artificial intelligence; ML: machine learning; MLP: multilayer perceptron; AUC: area under the curve; SES: socioeconomic status; CDS: clinical decision support; SDOH: social determinants of health; EHR: electronic health record; AE: asthma exacerbation; RF: random forest; LR: logistic regression; HOUSES: Housing-Based Socioeconomic Status; IQR: interquartile range, CP: computable phenotype; PPV: positive pressure ventilation; ICD-10: International Classification of Diseases, Tenth Revision; RUCA: Rural-Urban Commuting Area; OOB: out-of-bag; ESI: Emergency Severity Index

Author(s)	AI Model Type	Input Features Used	Outcome Predicted	Performance Metrics	Comparison with Traditional Methods
Emeryk et al., [13] (2023)	ML	- Wheezes, rhonchi, coarse and fine crackles intensity - Respiratory and heart rate - Inspiration-to-expiration ratio - SpO2 - PEF - Health survey responses	Asthma exacerbation detection	- AUC for wheezes (young children): 84% (95%CI: 82–85%) - AUC for rhonchi (older children): 81% (95%CI: 79–84%) - AUC for symptoms (survey) (adults): 92% (95%CI: 89–95%) - Highest AUC with combined parameters	AI-aided home stethoscope showed superior utility in exacerbation detection, particularly for pediatric patients compared to standard clinic-based monitoring
Harmon et al., [14] (2025)	MLP	Prehospital EMS data from 24,283 patient encounters (ages 2–18 years)	Pediatric asthma exacerbation (prehospital identification)	F1 Score: 0.95, Specificity: 1.00, Sensitivity: 0.91, NPV: 0.98, PPV: 1.00	Significantly outperformed rule-based CPs
Hurst et al., [15] (2022)	xgBoost	EHR, spatially resolved environmental data, temporally resolved climate, pollution, allergen, and influenza case data	Pediatric asthma exacerbation within 30–180 days	AUC: 0.730–0.742 (moderate); Sensitivity: 70%; PPV: 13.8% (180 days), 2.9% (30 days)	Not explicitly compared with traditional methods
Juhn et al., [16] (2022)	Existing machine learning models	EHR, including asthma severity, asthma diagnosis, HOUSES index	Asthma exacerbation prediction	BER across SES groups	NR
Overgaard et al., [17] (2022)	ML-based CDS System	Structured data and unstructured clinical notes (contextual data and supporting information)	Asthma exacerbation risk prediction	AUC = 0.8	Not explicitly compared
Rezaeiahari et al., [18] (2024)	Conditional Random Forest	- Prior-year asthma-related hospitalizations and ED visits - Total number of reliever and controller medications - Race and ethnicity - ICD-10 SDOH codes - RUCA codes - Child Opportunity Index	Asthma-related hospitalizations and emergency department visits in 2019	AUC: 73% (training), 72% (OOB) Sensitivity: 58% (training), 55% (OOB) Specificity: 77% (training), 78% (OOB)	NR
Seol et al., [19] (2021)	ML-based CDS Tool (A-GPS)	EHRs, clinical features related to asthma management	Risk of AE	- AE frequency: 12% (intervention) vs. 15% (control); OR: 0.82; 95% CI: 0.374–1.96; P=0.626- Time to review EHRs: 3.5 min vs. 11.3 min; P < 0.001- Healthcare costs: reduced in intervention group (not statistically significant; P = 0.12)	- No significant difference in AE frequency- Significantly reduced time for EHR review- Slight (non-significant) reduction in cost
Sills et al., [20] (2021)	AutoML	Data at ED triage, data one hour into ED visit, prior visit outcome, ESI level, time to first medication, time to triage	Need for hospitalization in pediatric ED	AUC: 0.914 (triage data), 0.942 (with additional data); Accuracy and F1 score also reported but values not stated	Outperformed RF (AUC: 0.831 & 0.886) and LR (AUC: 0.795 & 0.823)

Harmon et al. achieved exceptional results with an MLP model for prehospital pediatric asthma exacerbation identification, reporting an F1 score of 0.95, specificity of 1.00, and sensitivity of 0.91 [14]. This model significantly outperformed traditional rule-based computable phenotypes. In contrast, Hurst et al. found moderate performance (AUC: 0.730-0.742) when using EHR and environmental data for exacerbation prediction, noting that spatial data did not enhance model accuracy [15].

Juhn et al. identified socioeconomic bias in ML models, with worse predictive performance (measured by BER) in low-socioeconomic status (SES) children, suggesting that data incompleteness may contribute to AI bias [16]. Overgaard et al. developed an ML-based CDS system with strong predictive capability (AUC: 0.8), emphasizing the importance of contextual data and model explainability [17]. Rezaeiahari et al. reported moderate accuracy (AUC: 72-73%) for predicting asthma-related hospitalizations using administrative claims data, with prior hospitalizations and medication use as key predictors [18].

Seol et al. evaluated an ML-based CDS tool (assisted-global positioning system (A-GPS)) in a randomized clinical trial, finding no significant reduction in exacerbation frequency but demonstrating improved efficiency in EHR review time (3.5 vs. 11.3 minutes, p < 0.001) [19]. Sills et al. achieved high predictive performance (AUC: 0.914-0.942) using AutoML to forecast hospitalization needs in pediatric asthma patients, outperforming traditional models like random forest and logistic regression [20].

Results of Risk of Bias Assessment

The risk of bias assessment, conducted using the ROBINS-I tool for non-randomized studies and the Cochrane RoB 2 tool for the single randomized trial, revealed variability across the included studies. Among the non-randomized studies, those by Harmon et al. [14], Overgaard et al. [17], Rezaeiahari et al. [18], and Sills et al. [20] demonstrated low overall risk of bias, attributed to their use of large, standardized datasets, adjustments for key confounders, and objective outcome measurements. In contrast, the studies of Emeryk et al. [13] and Hurst et al. [15] were rated as having moderate risk, primarily due to uncontrolled age variability in the former and reliance on self-reported environmental data in the latter. Juhn et al.'s study was deemed high risk because of significant SES bias, a small sample size, and missing EHR data in low-SES groups, which likely skewed model performance [16]. The lone randomized trial, Seol et al., was assessed as low risk overall, with robust randomization and complete follow-up, though the lack of clinician blinding introduced a minor limitation [19]. These findings underscore the importance of addressing data completeness (e.g., missing EHR data [16]), confounding variables (e.g., environmental factors [15]), and algorithmic bias (e.g., SES disparities [16]) to enhance the reliability of AI applications in pediatric asthma research (Tables 3, 4).

**Table 3:** Risk of bias assessment for non-randomized studies (ROBINS-I Tool) ROBINS-I: Risk Of Bias In Non-randomized Studies of Interventions

Study (Author, Year)	Bias Due to Confounding	Bias in Participant Selection	Bias in Classification of Interventions	Bias Due to Missing Data	Bias in Outcome Measurement	Bias in Reported Results	Overall Risk of Bias
Emeryk et al., [13] (2023)	Moderate	Low	Low	Low	Moderate	Low	Moderate
Harmon et al., [14] (2025)	Low	Low	Low	Low	Low	Low	Low
Hurst et al., [15] (2022)	Moderate	Moderate	Low	Low	Moderate	Low	Moderate
Juhn et al., [16] (2022)	High	High	Low	High	Moderate	Low	High
Overgaard et al., [17] (2022)	Low	Low	Low	Low	Low	Low	Low
Rezaeiahari et al., [18] (2024)	Low	Low	Low	Low	Low	Low	Low
Sills et al., [20] (2021)	Low	Low	Low	Low	Low	Low	Low

**Table 4:** Risk of bias assessment for the randomized trial (Cochrane RoB 2 Tool)

Study (Author, Year)	Randomization Process	Deviations from Intended Interventions	Missing Outcome Data	Outcome Measurement	Selective Reporting	Overall Risk of Bias
Seol et al., [19] (2021)	Low	Low	Low	Low	Low	Low

Key Findings and Trends

The studies collectively underscored the potential of AI in pediatric asthma exacerbation prediction and management, with several recurring themes. First, multimodal data integration (e.g., clinical, environmental, and survey data) often enhanced model performance [13,15]. Second, ML models frequently outperformed traditional rule-based methods, particularly in large datasets [14,20]. Third, socioeconomic factors and data quality emerged as critical considerations, with biases observed in models trained on incomplete EHR data [16]. Finally, while AI tools showed promise in improving clinical efficiency (e.g., reduced EHR review time [19]), their impact on exacerbation frequency and patient outcomes remains an area for further investigation.

Discussion

The findings of this systematic review highlight the growing role of AI in predicting and managing pediatric asthma exacerbations, while also revealing critical challenges and opportunities for future research. The included studies demonstrated that AI models, particularly ML algorithms, can achieve moderate to high predictive accuracy for asthma exacerbations across diverse clinical settings. For instance, Emeryk et al. reported impressive AUC values (81-92%) using an AI-aided stethoscope combined with survey data, underscoring the potential of multimodal data integration [13]. Similarly, Harmon et al. achieved exceptional performance (F1 score: 0.95) with an MLP model for prehospital pediatric asthma identification, outperforming traditional rule-based methods [14]. These results align with broader trends in digital health, where AI is increasingly used to enhance diagnostic and predictive capabilities in chronic disease management [21]. However, the variability in model performance across studies suggests that the effectiveness of AI tools is highly context-dependent, influenced by factors such as data quality, sample size, and clinical setting.

One of the most compelling observations from this review is the superior performance of AI models over conventional methods in large datasets. Studies like that by Harmon et al. [14] and Sills et al. [20] demonstrated that ML algorithms (e.g., MLP, AutoML) could significantly outperform traditional statistical models, such as logistic regression, particularly when trained on comprehensive EHR data. This finding is consistent with prior research in other medical domains, where AI has shown promise in processing complex, high-dimensional data to identify patterns that may elude human clinicians or simpler models [22]. However, the review also revealed limitations in generalizability. For example, Hurst et al. found that incorporating spatial environmental data did not improve exacerbation prediction, suggesting that not all data types are equally valuable [15]. This contrasts with some studies in adult asthma, where environmental factors like air pollution have been strongly linked to exacerbation risk [23]. The discrepancy may reflect differences in pediatric populations or the specific methodologies used, emphasizing the need for pediatric-specific AI models.

A critical issue highlighted by this review is the impact of socioeconomic bias on AI performance. Juhn et al. identified significant disparities in predictive accuracy between high- and low-SES children, with worse performance in the latter group due to missing EHR data [16]. This aligns with growing concerns about algorithmic bias in healthcare AI, where underrepresented populations often receive suboptimal care due to data incompleteness or model training biases [24]. The findings underscore the importance of addressing data equity in AI development, as models trained on incomplete or biased datasets may perpetuate existing healthcare disparities. This is particularly relevant for asthma, a condition disproportionately affecting low-income and minority populations [25]. Future studies must prioritize inclusive data collection and bias mitigation strategies, such as adversarial debiasing or oversampling of underrepresented groups [26].

Another key theme is the role of AI in CDS systems. Overgaard et al. [17] and Seol et al. [19] explored ML-based CDS tools, with the former achieving strong predictive performance (AUC: 0.8) and the latter demonstrating significant efficiency gains in EHR review time (3.5 vs. 11.3 minutes). These results suggest that AI can streamline clinical workflows, reducing the burden on healthcare providers. However, Seol et al. found no significant reduction in exacerbation frequency, indicating that workflow efficiency does not always translate to improved patient outcomes [19]. This dichotomy mirrors broader debates in digital health about the trade-offs between process optimization and clinical efficacy [27]. While AI can enhance operational efficiency, its impact on long-term health outcomes remains uncertain, necessitating further research with patient-centered endpoints.

The review also identified gaps in model interpretability and usability. Overgaard et al. emphasized the importance of explainability in their ML-based CDS system, a feature often lacking in "black-box" AI models [17]. This is critical for clinician adoption, as healthcare providers are more likely to trust and use tools that offer transparent decision-making processes [28]. Similarly, Rezaeiahari et al. found that social determinants of health (SDOH) variables had minimal impact on their model’s performance, possibly due to poor data granularity or coding inconsistencies [18]. This contrasts with literature emphasizing the importance of SDOH in asthma outcomes [29], suggesting that future models should incorporate more robust SDOH metrics, such as neighborhood-level deprivation indices or housing quality data [30].

Despite the promise of AI, this review reveals several challenges in translating research findings into clinical practice. First, many studies relied on retrospective data, limiting their real-world applicability. For example, Hurst et al. [15] and Rezaeiahari et al. [18] used EHR and claims data, respectively, which may not capture the dynamic nature of asthma exacerbations. Prospective studies with real-time data collection are needed to validate these models in clinical settings. Second, the lack of standardization in outcome measures and performance metrics complicates cross-study comparisons. While AUC was commonly reported, other metrics like F1 scores or precision-recall curves were inconsistently used, making it difficult to assess model robustness comprehensively. This aligns with calls for standardized reporting guidelines in AI healthcare research [31].

Finally, the ethical implications of AI in pediatric asthma care warrant careful consideration. The high-risk bias identified in Juhn et al. [16] due to SES disparities raises concerns about equitable access to AI-driven interventions. Policymakers and developers must ensure that these tools do not exacerbate existing inequities, particularly for vulnerable populations. Additionally, the integration of AI into clinical practice requires rigorous regulatory oversight to ensure safety and efficacy, especially for pediatric applications where evidence is often extrapolated from adult studies [32].

Limitations

This systematic review has several limitations. First, the inclusion of only eight studies may limit the generalizability of the findings, although the diverse methodologies and settings provide valuable insights. Second, the predominance of studies based in the United States may introduce geographic bias, as healthcare systems and data availability vary globally. Third, the reliance on AUC as the primary performance metric may overlook other clinically relevant measures, such as positive predictive value or calibration. Finally, the review did not assess publication bias, which could skew the results toward positive findings.

## Conclusions

This study highlights the potential of AI to transform pediatric asthma care through improved prediction and management of exacerbations. The studies demonstrate that ML models can outperform traditional methods, particularly when leveraging multimodal data and large datasets. However, challenges such as socioeconomic bias, data incompleteness, and limited real-world validation must be addressed to ensure equitable and effective implementation. Future research should prioritize prospective studies, standardized reporting, and bias mitigation strategies to unlock the full potential of AI in pediatric asthma care. By addressing these gaps, AI can move from a promising tool to a clinically impactful solution for children with asthma.
